# Biomarkers and the Role of α-Synuclein in Parkinson’s Disease

**DOI:** 10.3389/fnagi.2021.645996

**Published:** 2021-03-23

**Authors:** Tingting Du, Le Wang, Weijin Liu, Guanyu Zhu, Yingchuan Chen, Jianguo Zhang

**Affiliations:** ^1^Department of Functional Neurosurgery, Beijing Neurosurgical Institute, Capital Medical University, Beijing, China; ^2^Molecular Biology Laboratory for Neuropsychiatric Diseases, Department of Neurobiology, Beijing Institute of Brain Disorders, Capital Medical University, Beijing, China; ^3^Key Laboratory for Neurodegenerative Disease of the Ministry of Education, Key Laboratory of Neural Regeneration and Repair, Beijing Key Laboratory on Parkinson’s Disease, Department of Neurobiology, School of Basic Medical Sciences, Capital Medical University, Beijing, China; ^4^Department of Neurosurgery, Beijing Tiantan Hospital, Capital Medical University, Beijing, China; ^5^Beijing Key Laboratory of Neurostimulation, Beijing Municipal Science and Technology Commission, Beijing, China

**Keywords:** Parkinson’s disease, α-synuclein, biomarker, genetics, immune inflammation, imaging

## Abstract

Parkinson’s disease (PD) is a progressive neurodegenerative disorder characterized by the presence of α-synuclein (α-Syn)-rich Lewy bodies (LBs) and the preferential loss of dopaminergic (DA) neurons in the substantia nigra (SN) pars compacta (SNpc). However, the widespread involvement of other central nervous systems (CNS) structures and peripheral tissues is now widely documented. The onset of the molecular and cellular neuropathology of PD likely occurs decades before the onset of the motor symptoms characteristic of PD, so early diagnosis of PD and adequate tracking of disease progression could significantly improve outcomes for patients. Because the clinical diagnosis of PD is challenging, misdiagnosis is common, which highlights the need for disease-specific and early-stage biomarkers. This review article aims to summarize useful biomarkers for the diagnosis of PD, as well as the biomarkers used to monitor disease progression. This review article describes the role of α-Syn in PD and how it could potentially be used as a biomarker for PD. Also, preclinical and clinical investigations encompassing genetics, immunology, fluid and tissue, imaging, as well as neurophysiology biomarkers are discussed. Knowledge of the novel biomarkers for preclinical detection and clinical evaluation will contribute to a deeper understanding of the disease mechanism, which should more effectively guide clinical applications.

## Introduction

Parkinson’s disease (PD) is a complex, chronic, and progressive neurodegenerative disease characterized by the presence of Lewy bodies (LBs) in vulnerable populations of neurons. The major component of LBs is insoluble α-synuclein (α-Syn) fibrils (Spillantini et al., 1997). The etiology of PD is likely multifactorial, and involves the interplay among aging, genetic susceptibility, and environmental factors (Pang et al., 2019); however, the α-Syn protein is a central component in the pathogenesis of the disease. Accumulating evidence suggests that α-Syn oligomers play a significant role in the PD neurodegenerative process by impairing many subcellular functions (Colla et al., 2012; Choi et al., 2013). Generally, degeneration of approximately 70% of dopaminergic (DA) neurons in the substantia nigra (SN) pars compacta (SNpc) of the midbrain region takes place before the clinical symptoms occur (Postuma et al., 2010; Heisters, 2011). According to clinical and imaging findings, the neurodegenerative process of PD may begin 7–10 years before the appearance of the classic motor symptoms of bradykinesia, rigidity, and tremors (Hawkes, 2008; Schapira and Tolosa, 2010). PD is diagnosed from motor symptoms, but non-motor symptoms are often manifest during a prolonged prodromal phase as much as 20 years before the onset of the motor features (Kalia and Lang, 2015; Armstrong and Okun, 2020). Treatment can therefore only be initiated in the late phase of the disease. Although considerable progress has been made in understanding its pathogenesis, early and accurate diagnosis of PD is still a formidable challenge (Postuma et al., 2016). This is largely a result of the lack of well-established biomarkers for early diagnosis and monitoring of progression.

Biomarkers are required to detect the disease in the early stages when prevention is possible. Various biomarkers providing early diagnosis of the disease include clinical, imaging, pathological, physiological, biochemical, and genetic parameters. Moreover, biomarkers—alone or in combination—are used to diagnose and monitor the evolution of PD. This review article describes various biomarkers available for PD, discusses recent advances in their development, and focuses on the roles of α-Syn in the neurodegenerative process during PD.

## Genetic Biomarkers

### SNCA

PD is typically a sporadic disease, but in a small proportion of patients, mutation of a dominantly or recessively inherited gene can cause PD. Although relatively rare, these genetic forms of PD have revealed much about the molecular pathogenesis of sporadic PD (Kalia and Lang, 2015). The first gene identified was *SNCA* (Polymeropoulos et al., 1997), which encodes α-Syn. *SNCA* mutations are associated with autosomal dominant PD. *SNCA* mutations as a genetic cause of PD led to the identification of α-Syn as a major component of LBs and neurites (Spillantini et al., 1997). In particular, α-Syn is implicated in the pathogenesis of PD, because it is a major component of LBs. The aggregation of α-Syn and its incorporation as a major component of LBs is the hallmark of PD. Approximately 90% of insoluble α-Syn in LBs is phosphorylated, and phosphorylation at Ser-129 (a major phosphorylation site) is characteristic of PD (Chen and Feany, 2005). Additionally, point mutations in the *SNCA* gene lead to familial PD (Polymeropoulos et al., 1997), as do relatively rare duplications or triplications of the *SNCA* locus, which result in elevated α-Syn in both the brain and blood (Miller et al., 2004). Point mutations, duplications, and triplications in the *SNCA* gene cause PD with high penetrance, and some SNPs of this gene have been identified as genetic risk factors for PD (Dekosky and Marek, 2003; Gasser, 2009). Highly penetrant familial mutations or multiplications in the *SNCA* have been shown to cause aggressive early-onset PD. Additionally, a common SNP in the *SNCA* gene that is enriched in PD patients has been shown to increase expression *in vitro* (Mizuta et al., 2013). Moreover, variants in the gene have been identified as a risk factor in Genome–Wide Association Studies (GWAS; Nalls et al., 2011). Thus, α-Syn in blood, plasma, or cerebrospinal fluid (CSF) is a promising candidate as a biomarker for diagnosing PD.

The first evidence showing that *SNCA* gene deregulation is directly associated with PD came from familial PD cases, where point mutations at the 53rd (alanine to threonine), 30th (alanine to proline), and the 46th (glutamine to lysine) amino acids were discovered, which led to an autosomal dominant form of disease inheritance along with early-onset (Polymeropoulos et al., 1997; Krüger et al., 1998; Zarranz et al., 2004). Both duplication and triplication of *SNCA* were strongly associated with severity and early onset in familial PD (Singleton et al., 2003; Chartier-Harlin et al., 2004). Several SNPs identified throughout *SNCA* were found to interfere with the normal transcriptional regulation of the gene and the binding of microRNA. The study revealed that the expression levels of microRNA miR-19b were downregulated in patients with idiopathic rapid eye movement sleep behavior disorder, and antedated the diagnosis of PD after 4.67 ± 2.61 years of follow-up (Fernández-Santiago et al., 2015). It, therefore, has the potential for early diagnosis of PD. Locascio et al. reported a consistent association of reduced *SNCA* transcripts in accessible peripheral blood and early-stage PD in 863 patients and suggested a clinical role as a potential predictor of cognitive decline (Locascio et al., 2015). At present, the most studied epigenetic-based biomarker for PD is DNA methylation in the *SNCA* gene. One report showed a 2-fold decrease in global DNA methylation in the cortex of human postmortem PD samples, with significant hypomethylation of the *SNCA* intron1 region (Desplats et al., 2011). Efforts have therefore considered using *SNCA* or some other PD-implicated gene methylation in peripheral tissues as a biomarker for PD (Jakubowski and Labrie, 2017). However, α-Syn, and subsequently LBs, are markers of other neurodegenerative diseases collectively termed α-synucleinopathies, a group that includes PD (with or without dementia), dementia with LBs, an LB variant of Alzheimer’s disease, and multiple system atrophy (MSA). Therefore, a combination of imaging, biochemical analyses, and genetic biomarkers might be required.

### Other Genes

In the past two decades, substantial progress has been made in the genetics of PD. Data from Mendelian genetics and GWAS have identified several chromosomal loci that cause or regulate the risk of PD (Hernandez et al., 2016; Deng et al., 2018). Genetic factors are associated with specific clinical pictures and may influence the response to therapy. Mutations of *SNCA*, Leucine-rich repeat kinase 2 (*LRRK2*), *Parkin*, PTEN-induced putative kinase 1 (*PINK1*), and *DJ-1* genes have been associated with monogenic forms of PD and linked to specific clinical characteristics slightly different from those observed in non-mutated patients (Koros et al., 2017).

Since the discovery of *SNCA* mutations as a cause of PD, five additional genes have been proposed to mediate autosomal dominant forms of PD: *LRRK2*, *VPS35*, *EIF4G1*, *DNAJC13*, and *CHCHD2* (Kalia and Lang, 2015). Mutations in *LRRK2* are the most common causes of dominantly inherited PD (Corti et al., 2011) because they are found in approximately 4% of familial PD patients and account for 1% of sporadic PD worldwide. The most common *LRRK2* mutation results in a Gly2019Ser amino acid substitution (Healy et al., 2008). *Parkin*, *PINK1*, and *DJ-1* are associated with autosomal recessive forms of PD (Kalia and Lang, 2015). Mutations in *Parkin* are the most common cause of autosomal recessive PD. In patients with PD onset before the age of 45 years, *Parkin* mutations are seen in up to 50% of familial cases and about 15% of sporadic cases (Periquet et al., 2003). Patients with mutations in *Parkin* usually show juvenile PD (JPD, onset <21 years old) or early-onset PD (between 21 and 40 years old) with a slow clinical course, excellent response to low doses of levodopa, and frequent treatment-induced dyskinesias (Lohmann et al., 2009). Mutations in *PINK1* and *DJ-1* are less common causes (1–8% and 1–2% of early-onset sporadic PD, respectively; Singleton et al., 2013). *DJ-1*, also known as Parkinsonism-associated deglycase encoded by the *PARK7* gene, is a multifunctional protein that plays a neuroprotective role in oxidative stress during neurodegeneration (Saito, 2014). An increase of DJ-1 ultimately reflects oxidative stress in PD patients, making it a biomarker for PD. However, the DJ-1 level is very high in erythrocytes, and in the CSF and plasma, it is affected by hemolysis and contamination by erythrocytes (Saito, 2014). Therefore, when detecting DJ-1 level in the CSF and plasma, an evaluation of hemolysis and contamination by erythrocytes is very necessary. Generally, autosomal dominant PD tends to have an age of onset similar to sporadic PD, while recessively inherited Parkinsonism is more frequently associated with early onset (age <40 years; Schrag and Schott, 2006).

The greatest genetic risk factor for developing PD is a mutation in *GBA*, which encodes β-glucocerebrosidase (GCase), the lysosomal enzyme deficient in Gaucher disease (GD) (Sidransky and Lopez, 2012). The results of a large multicenter study of more than 5,000 patients with PD and an equal number of matched controls showed an odds ratio >5 for any *GBA* mutation in PD patients vs. control subjects (Sidransky et al., 2009). PD patients with some *GBA* mutations (GBA-PD) tend to have an earlier onset of symptoms, more aggressive disease, with more severe and earlier cognitive impairments and psychiatric manifestations (Balestrino and Schapira, 2018). One study reported that GCase activity was significantly lower in GBA-PD than in idiopathic PD patients, and even lower in GD-PD patients, suggesting that GCase activity could be a possible marker of mutated *GBA* (Ortega et al., 2016). Deficiency of GCase increases the risk of Parkinsonism, which appears to be driven by the direct effect of GCase deficiency and lysosomal dysfunction on α-Syn aggregation. The GCase activity in the CSF could therefore further serve as a PD biomarker (Magdalinou et al., 2014). Additional genes associated with Parkinsonism identified from relatives or patient cohorts include *ATP13A2*, *C9ORF72*, *FBXO7*, *PLA2G6*, *POLG1*, *SCA2*, *SCA3*, *SYNJ1*, and *RAB39B*. Parkinsonism due to mutations in these genes is quite rare and usually associated with features atypical for PD (e.g., prominent cognitive impairment, ophthalmological abnormalities, pyramidal signs, or ataxia; Kalia and Lang, 2015).

## Immune Inflammatory Biomarkers

### Immune Inflammation in PD

Under physiological conditions, the brain provides an immunosuppressive environment to maintain the function of the central nervous systems (CNS). When this environment is destroyed, such as in infectious diseases, autoimmune diseases, or neurodegenerative diseases, a series of immune responses are activated, sometimes even leading to chronic inflammation. Although it has been shown that various cells are involved, including peripheral immune cells, neurons, microglia, and astrocytes that infiltrate the brain tissue, the role of microglia is most conspicuous as resident macrophages of the CNS. Microglia are in the “resting or inactive” state under physiological conditions; however, almost any form of disturbance of the CNS microenvironment, such as infection, injury, and aging, can result in microglial activation.

Since the discovery of activated microglia in the SNpc of PD patients in 1988, increasing attention has been directed toward the study of neuro-immunoinflammatory pathogenesis of PD, characterized by microglial activation. Autopsies of PD patients have shown that the loss of DA neurons is accompanied by microglial activation, an inflammatory response, and the expressions of proinflammatory cytokines in microglia, such as TNF-α, IFN-γ, and IL-1β (Mirza et al., 2000). The early positron emission tomography (PET) results of PD patients have shown that the number of activated microglia was significantly increased, accompanied by the loss of DA neurons in the midbrain (Ouchi et al., 2005), which suggested that the neuroinflammatory response caused by excessive microglial activation had a significant impact on the progression of DA neuron degenerative in PD. These studies also suggest that assessing microglial activation in the substantia nigra (SN), as well as DA neuron injury by imaging techniques such as PET, can be an early diagnostic biomarker of PD.

A large number of activated microglia have also been found in the 1-methyl-4-phenyl 1,2,3,6-tetrahydropyridine (MPTP), rotenone, and 6-hydroxydopamine (6-OHDA) models and the activation of microglia can enhance their neurotoxicity to DA neurons, suggesting that microglial activation may be one of the main factors promoting the death of DA neurons (Zheng et al., 2008; Sui et al., 2009; Walsh et al., 2011). The systemic inflammation induced by IL-1β and LPS can exacerbate the death of DA neurons in PD animal models (Pott Godoy et al., 2008; Byler et al., 2009). However, some anti-inflammatory drugs, such as ibuprofen and minocycline, can reduce the inflammatory effects, lower microglia activation, and alleviate the degeneration of DA neurons in the SN in PD animal models (Wu et al., 2002; Zheng et al., 2008; Walsh et al., 2011), which suggests that suppressing microglial activation and proinflammatory cytokine release can inhibit neurodegeneration. Also, transgenic animal models have confirmed the association between microglia and PD (Lachenmayer and Yue, 2012; Deng and Yuan, 2014), in which microglia were activated and TNF-α secretion was increased in the SN of mice overexpressing α-Syn, with subsequent neuronal death (Su et al., 2008; Sanchez-Guajardo et al., 2010). Both transgenic and inflammatory PD models can explain some pathological changes of the disease, but they cannot fully explain the role of innate immunity and the slow onset of degenerative diseases; however, these studies fully demonstrate the involvement of immune inflammation in PD pathogenesis.

Activation of the immune system can affect the synthesis and secretion of many soluble immune factors, including cytokines, chemokines, and complement, which are significantly increased in the PD postmortem brain (Reale et al., 2009; Grozdanov et al., 2014). These increased cytokines are mainly present in the nigrostriatal system rather than in other brain regions, suggesting that they are produced only at the sites of DA neuron damage. Furthermore, the level of cytokines in CSF as a possible marker of early diagnosis or disease progression in PD is worthy of study and attention. In our opinion, the content of cytokines in CSF can be used as a marker for the progression of PD and can provide a target for PD treatment. However, a comprehensive assessment of PD should also be performed by including other biomarkers. Although microglial activation and neuroinflammation may provide directions and ideas for PD pathogenetic mechanisms and therapeutic strategies, it remains to be clarified whether microglial activation is the result or cause of DA neuron degeneration.

### The Role of α-Syn in Immune Inflammation of PD

The α-Syn aggregates are thought to be the major component of PD LBs, and α-Syn is found both in the cytoplasm but also in intercellular space. Aberrant accumulation of α-Syn can promote both neuronal dysfunction and neuroinflammation. Microglia act as the resident immune cells of the brain, which closely monitor changes of the microenvironment and maintain microenvironment homeostasis. Experimental evidence has shown that α-Syn aggregates can be surrounded by microglia, and microglia can be activated by extracellular α-Syn aggregates, leading to increased extracellular superoxide, inflammatory cytokines, and the selective loss of DA neurons (Couch et al., 2011). Some studies have also shown that soluble α-Syn can also activate microglia (Klegeris et al., 2008).

Microglia can phagocytose extracellular α-Syn aggregates, which can be degraded by the autophagosome. Additionally, the Fc-γ receptor is required for uptake of extracellular, aggregated α-Syn and neuroinflammation induced by α-Syn (Cao et al., 2012). Fc-γ receptor knockout has been shown to attenuate the immune response, microglia activation, and DA neuron neurodegeneration, which is triggered by adeno-associated virus serotype 2 to selectively overexpress human α-Syn in the SN (Cao et al., 2010). The Fc-γ receptor^−/−^ microglia do not exhibit an inflammatory response to aggregated α-Syn, suggesting that phagocytosis of α-Syn into microglia is required for inducing neuroinflammation and neurodegeneration in PD patients (Cao et al., 2012). Apart from Fc-γ receptor-mediated phagocytosis of α-Syn, toll-like receptors (TLRs) are also targeted by α-Syn treatment. The α-Syn can act as a damage-associated molecular pattern, which activates TLRs, and it may be an important mediator of neuroinflammation in PD patients. TLR2 and TLR4 expressions increase in postmortem brain tissue from PD patients (Drouin-Ouellet et al., 2014; Dzamko et al., 2017), and TLR2 is strongly localized to α-Syn positive LBs (Dzamko et al., 2017). Furthermore, both treatments with blocking antibody against TLR2 and depletion of TLR2 in microglia result in the elimination of cytokine release induced by conditioned medium from SH-SY5Y cells overexpressing α-Syn (Kim et al., 2013). Microglial phagocytic activity, proinflammatory cytokine release, and reactive oxygen species (ROS) production triggered by recombinant α-Syn treatment are downregulated in the TLR4 deficient murine (Fellner et al., 2013). The current findings suggest that TLR2 and TLR4 play a modulatory role in microglial activation and proinflammatory responses triggered by α-Syn in PD.

There is evidence that α-Syn can be secreted into the extracellular environment, and most of the extracellular α-Syn is aggregated, misfolded, and increased in amount. This extracellular α-Syn activates microglia and the immune system, to induce inflammatory responses during the pathogenesis of PD. Studies have assessed the effect of oligomeric or fibrillar α-Syn on microglia, and have shown that an increase in TNF-α (Daniele et al., 2015; Pradhan et al., 2017; Hughes et al., 2019), IL-1β (Daniele et al., 2015; Pradhan et al., 2017), IL-6 (Acuña et al., 2019), COX-2, and iNOS (Pradhan et al., 2017), as well as an increase in ROS or NO (Daniele et al., 2015; Pradhan et al., 2017; Panicker et al., 2019), are involved in neurotoxicity. Aggregated α-Syn also has been found to increase inflammasome activation and even pyroptosis. Fibrillary α-Syn in mouse microglia results in a delayed but robust activation of NLRP3 inflammasomes, leading to extracellular IL-1β and caspase recruitment domain release (Zhou et al., 2016; Gordon et al., 2018). Furthermore, phosphorylated and nitrated α-Syn also activates microglia, increases proinflammatory cytokines release, and induces inflammation (Reynolds et al., 2009; Christiansen et al., 2016; Duffy et al., 2018). Also, *in vitro* studies using different doses of monomeric recombinant α-Syn have stimulated both primary cultured microglia and microglia/macrophage cell lines, and have found that extracellular α-Syn can induce a pro-inflammatory response in microglia with an increase in proinflammatory cytokines (Boza-Serrano et al., 2014; Hu et al., 2016; Rabenstein et al., 2019; Shao et al., 2019; Wang L. et al., 2020), as well as an increase in reactive oxygen or COX-2 (Lee et al., 2010; Boza-Serrano et al., 2014; Hu et al., 2016), associated with inflammation. These results confirm that extracellular α-Syn has an important role in chronic inflammation during the pathogenesis of PD.

Microglia have stronger phagocytosis of extracellular α-Syn when compared to other cells in the CNS (Zhang et al., 2005), but different groups have found this phagocytic activity is different for different species of α-Syn. Microglia incubated with both wild-type and A53T α-Syn showed increased phagocytic activity, but an opposite effect was found in A30P and E46K α-Syn (Roodveldt et al., 2010). Also, monomeric α-Syn-stimulated microglia upregulated phagocytosis, while aggregated α-Syn inhibited both basal and LPS-induced phagocytosis (Park et al., 2008). Also, microglial phagocytic activity is increased after fibrillary α-Syn treatment dependent on TLR4 (Fellner et al., 2013). In contrast, microglia-overexpressed A30P or A53T α-Syn showed a pro-inflammatory response and impaired phagocytosis (Rojanathammanee et al., 2011). However, microglia from α-Syn knock-out mice showed increased basal levels of proinflammatory cytokines, increased expression of CD68, and impaired phagocytic ability, when compared with wild-type microglia, suggesting that the levels of α-Syn may be related to phagocytosis.

During the progression of PD, extracellular α-Syn can undergo phagocytosis by microglia, leading to microglial activation, but the ability of activated microglia to degrade α-Syn is reduced, with increased α-Syn accumulation in the cytoplasm, which causes continuous activation of microglia, and eventually leads to chronic inflammation and long-term damage to DA neurons.

## Fluid and Tissue Biomarkers

### The α-Syn Species in CSF and Blood

As early as 2003, El-Agnaf and his colleagues first reported the identification of α-Syn in human CSF (El-Agnaf et al., 2003). Since then, different immunoassays for CSF α-Syn detection have been compared in different laboratories, and most studies suggested that CSF α-Syn is lower in PD patients (Eusebi et al., 2017; Parnetti et al., 2019). Reports on serum and plasma quantitative detection of α-Syn have provided ambiguous results, whether higher, lower, or not significantly different in PD patients when compared with controls (Ding et al., 2017; Lin et al., 2017; Chang et al., 2019; Fayyad et al., 2019). Several studies provide strong evidence that soluble α-Syn oligomers (o-α-Syn), which have been proven to be more toxic, have been commonly found at higher levels in the CSF and plasma of PD patients (Kayed et al., 2003; El-Agnaf et al., 2006). Furthermore, it has been reported that the ratio of CSF o-α-Syn/total α-Syn could improve the sensitivity and specificity of PD diagnosis (Parnetti et al., 2014). As for the detection of α-Syn aggregates, two highly specific and sensitive methods, protein-misfolding cyclic amplification (PMCA) and real-time quaking-induced conversion (RT-QuIC) have been recently developed, and higher levels of α-Syn aggregates have been detected in the CSF samples of PD patients (Shahnawaz et al., 2017; Rossi et al., 2020). Shahnawaz et al. (2020) also used PMCA and found distinctly aggregated α-Syn strains in the CSF from PD and MSA patients, with a sensitivity reaching 95.4%. Taken together, the results show that different forms of α-Syn, especially o-α-Syn and misfolded α-Syn aggregates, detected by ultrasensitive techniques, are promising markers for early diagnosis and differential diagnosis of PD.

The α-Syn phosphorylation at serine-129 (pSer129 α-Syn, p-α-Syn) has also been shown to be abnormally elevated in CSF and plasma of PD patients (Foulds et al., 2011; Landeck et al., 2016; Majbour et al., 2016). Furthermore, Lin et al. (2019) found that plasma p-α-Syn correlated with motor severity and progression in PD patients. Notably, p-α-Syn is relatively unstable due to environmental factors. Cariulo et al. recently used ultrasensitive immunoassays based on single-molecule counting technology and found that p-α-Syn in CSF was undetectable in PD patients, even though plasma p-α-Syn could be easily detected (Cariulo et al., 2019). These results showed that p-α-Syn detection still lacked further validation in independent laboratories, so it is recommended that phosphatase inhibitors be added to samples at the time of collection. In general, studies on α-Syn still lack confirmation, as immunoassays and standardized antigens for quantification have varied from different laboratories. Besides, red blood cell (RBC) contamination also limits the usefulness of CSF and blood α-Syn species measurements. A standardized process for sample collection and experimental analyses are therefore still needed for an accurate diagnosis of PD.

### Other Biomarkers in CSF and Blood

In addition to α-Syn species, studies have also focused on searching for other PD biomarkers. Lamontagne-Proulx et al. (2019) found that extracellular vesicles (EV) in the plasma of PD patients contained a specific signature of proteins. These features could reliably differentiate control subjects from mild and moderate PD patients, which suggested that EV blood-based assays have the potential to be used as PD biomarker assays (Lamontagne-Proulx et al., 2019). In another study, Youn et al. (2018) found that the levels of LC3B, Beclin1, and LAMP-2 were higher in the CSF of PD patients, which means that CSF levels of some autophagy-related proteins might represent potential diagnostic and prognostic biomarkers of early-stage PD in patients (Youn et al., 2018). Also, Kim et al. (2019) evaluated the levels of β-amyloid 1–42 (Aβ1–42), α-Syn, tau, and phosphorylated tau181 in the CSF of PD patients, during a median follow-up of 4 years; only Aβ42 among these biomarkers were associated with the development of a freezing gait (Kim et al., 2019). Moreover, Posavi et al. (2019) used multicohort proteomics analyses to select blood-based biomarkers for PD patients. Their results showed that bone sialoprotein, osteomodulin, aminoacylase-1, and growth hormone receptor robustly associated with PD across multiple clinical sites (Posavi et al., 2019). Recently, a review showed that CSF and blood levels of amino acids might also become potential biomarkers for PD. They found decreased CSF levels of glutamate and taurine and increased CSF levels of tyrosine; decreased serum/plasma levels of aspartate, serine, tryptophan, and lysine, and increased serum/plasma proline and homocysteine levels (Jiménez-Jiménez et al., 2020). Taken together, these markers, and many other proteins such as circulating microRNAs and neurofilament light (He et al., 2018; Khalil et al., 2018; van den Berg et al., 2020; Wang H. et al., 2020), have all been reported to be associated with PD. However, Dos Santos et al. (2018) has also proposed that the potential biomarkers lacked robustness and reproducibility in supporting diagnosis in the early clinical stages of PD; therefore, we still need further validation to confirm these findings.

### The α-Syn Species in RBCs

RBCs are the major source of α-Syn in peripheral blood cells (Barbour et al., 2008). In the last several years, expanded insights into PD had revealed some relationships between PD and RBC-α-Syn. Wang et al. found that the ratio of o-α-Syn/total RBC protein was higher in PD patients than in the control groups. However, there was no correlation between RBC o-α-Syn levels and the Unified Parkinson’s disease Rating Scale (UPDRS) motor scale score or progression of motor degeneration (Wang et al., 2015). In another study, Abd-Elhadi et al. (2015) found that the ratio of α-Syn/proteinase K-resistant α-Syn was significantly lower in PD patients when compared with healthy controls. Post-translational modifications (PTMs) of RBC-α-Syn have also been identified. Vicente Miranda et al. (2017) found that the levels of phosphorylated Y125, nitrated Y39, and glycated α-Syn were increased in RBCs from PD patients, while SUMOylated α-Syn was reduced. Moreover, these PTMs correlated with disease severity and duration. The combinatorial analyses of these PTMs in RBCs resulted in increased sensitivity in clinical trials (Vicente Miranda et al., 2017). Notably, there was a significant difference between the RBC-α-Syn from the membrane and cytosolic fractions. Zhang et al. (2005) found that, compared to healthy controls, the levels of total and aggregated α-Syn were significantly higher in the membrane fraction, but not in the cytosolic component of PD patients. Furthermore, the level of p-α-Syn was higher in PD patients in the cytosolic fraction, while to a lesser extent, it was also higher in the membrane fraction (Tian et al., 2019). Papagiannakis et al. (2018) also collected erythrocyte membranes and detected higher levels of α-Syn dimers and the dimer to monomer ratio in mutant carriers of the *GBA* gene (GBA-PD) and PD patients without known mutations (Papagiannakis et al., 2018). It is noteworthy that different forms of RBC-α-Syn also changed in GD and patients with MSA (Moraitou et al., 2016; Liu et al., 2019). It was also reported that there was no significant difference in the o-α-Syn/total protein ratio in RBCs between PD and MSA patients (Wang et al., 2015). Thus, further research is still needed to determine if RBC-α-Syn can be used for differential diagnosis of PD.

### The α-Syn Species in Peripheral Tissues

Stokholm et al. (2016) found that gastrointestinal deposits of p-α-Syn were found in 56% of patients with prodromal PD when compared with 26% of control subjects, and they detected Lewy pathology in the gastrointestinal tract of patients up to 20 years before their diagnoses (Stokholm et al., 2016). Tolosa and co-workers also found aggregated α-Syn in submandibular gland tissues in patients with idiopathic rapid eye movement sleep behavior disorder (Vilas et al., 2016). Since then, studies have been conducted to detect abnormally aggregated α-Syn in multiple peripheral tissues and biofluids of PD patients (Tsukita et al., 2019; Chahine et al., 2020).

Peripheral biofluid samples such as saliva, tears, and urine are commonly used for clinical testing and diagnosis. Bougea et al. (2019) summarized the use of salivary α-Syn as a diagnostic biomarker for PD. They found that the potential use of salivary o-α-Syn but not total α-Syn was more promising (Bougea et al., 2019). In addition to o-α-Syn in salivary glands, Cao et al. (2019) reported that extracellular vesicles from PD saliva also obtained higher levels of o-α-Syn and higher o-α-Syn/α-Syn ratios (Cao et al., 2019). It was also suggested that salivary miR-153 and miR-223 levels may serve as non-invasive diagnostic biomarkers of idiopathic PD (Chahine et al., 2020). In addition to saliva, studies have also found higher levels of o-α-Syn in basal tears of PD patients (Hamm-Alvarez et al., 2019). Recently, Nam et al. found distinct forms of o-α-Syn in urine, which might be useful for PD diagnosis (Nam et al., 2020). In addition to these biological fluids, needle biopsies for labial minor salivary glands and submandibular glands, and detection of the deposition of α-Syn or p-α-Syn in PD patients are also possibilities for clinical applications (Adler et al., 2017; Iranzo et al., 2018; Shin et al., 2019). Furthermore, biopsy samples of submandibular glands were reported to have increased sensitivity and specificity, when compared to minor salivary glands (Campo et al., 2019). Manne et al. (2020) recently used the RT-QuIC technique to quantify pathological α-Syn level in paraffin-embedded submandibular glands of PD patients and healthy controls, where the sensitivity and specificity reached 100% and 94%, respectively (Manne et al., 2020).

Skin biopsy is also a promising diagnostic tool for PD. As early as 2014, Donadio et al. evaluated the skin biopsies from proximal and distal sites of small nerve fibers from idiopathic PD patients. They found that p-α-Syn was deposited in the proximal cervical skin, while nothing was detected in controls (Donadio et al., 2014). In subsequent studies, they also detected p-α-Syn both in proximal and distal sites of small nerve fibers from dementia with LB patients (Donadio et al., 2017). Similarly, it was reported that E46K-*SNCA* carriers had p-α-Syn aggregates in intraepidermal nerve fibers, and the severity of the latter skin abnormalities was correlated with sudomotor dysfunction in hands (Carmona-Abellan et al., 2019). Kuzkina et al. (2019) used conformation-specific antibodies and digestion with proteinase K to show that there was no obvious difference between α-Syn deposits in dermal cutaneous nerve fibers and midbrains of PD patients. In addition to early diagnosis of PD, abnormally aggregated α-Syn in skin nerve fibers might also be used for differential diagnosis with other diseases. Donadio et al. (2020) recently reported that the distribution difference of p-α-Syn in cutaneous nerves might help to distinguish MSA Parkinsonism type from PD with orthostatic hypotension. Wang et al. also used RT-QuIC/PMCA and found that skin α-Syn had aggregation seeding activity that was significantly higher in individuals with PD and other synucleinopathies, compared with those with tauopathies and non-neurodegenerative controls (Wang Z. et al., 2020).

In general, these results suggest that abnormally aggregated α-Syn in peripheral tissues and biofluids might be used for early diagnosis and differential diagnosis for PD. The development of new technologies, such as RT-QuIC and PMCA, has greatly assisted in these studies. However, it is also noteworthy that standardized methods for sample collection and detection are very important in the entire process, and related studies are still needed for further validations.

## Imaging Biomarkers

Imaging biomarkers have been increasingly used in the diagnosis of PD, to provide support for clinical observations. Neuroimaging using magnetic resonance imaging (MRI), positron emission tomography (PET), and transcranial sonography (TCS) can provide important information on brain structure and function in PD, and can also serve as an adjunct to clinical assessments. Imaging has been used to differentiate PD from other movement disorders, and to facilitate diagnostic accuracy.

### MRI

Traditional MRI is usually used to exclude secondary Parkinsonism, including neoplasms, vascular Parkinsonism, and multiple sclerosis. However, in recent years, new studies have reported that brain regions are widely involved in patients with PD. With the development of MRI, multiple sequences of MRI have been used to investigate change during PD.

T1-weighted (T1) and T2-weighted (T2) structural MRI has been used to estimate the cortical thickness and structural volume. Gerrits et al. (2016) performed voxel-based morphometry on PD patients to investigate the gray matter volume, and found that PD patients showed cortical thinning in the left pericalcarine gyrus, extending to the cuneus, precuneus, lingual areas, left inferior parietal cortex, bilateral rostral middle frontal cortex, and right cuneus, and increased cortical surface areas in the left pars triangularis (Gerrits et al., 2016). Lyoo et al. (2011) reported that the parieto-temporal associated cortex cortical thickness was negatively correlated with disease duration, total UPDRS motor score, and bradykinesia and axial motor deficit subscores. The SN plays an indispensable role in the pathology of PD (Lehéricy et al., 2014). Volumetric differences of SN have been investigated in some studies using MRI, with varying results. Minati et al. (2007) reported that loss of the SN was observed in PD patients, when compared with control subjects, nevertheless, some studies did not observe any volumetric differences of SN in PD patients (Oikawa et al., 2002; Péran et al., 2010). Also, a study performed 7T MRI of PD patients, and found higher volumes of the SN in PD patients, when compared with the unsmooth boundary between the SN and crus cerebri (Kwon et al., 2012). Cosottini et al. (2014) found that three-dimensional susceptibility-weighted 7T-MRI showed a three-layered organization of the SN, which allowed discrimination between PD patients and control subjects with a sensitivity and specificity of 100% and 96.2%, respectively (Cosottini et al., 2014).

Quantitative susceptibility mapping (QSM), which compensates for the nonlocality of magnetic field distribution, could provide a robust estimation of magnetic susceptibility that is correlated with the iron content of the brain. PD patients were divided into Hoehn–Yahr stage ≤2.5 and Hoehn–Yahr stage ≥3 into early-stage and late-stage PD groups. The QSM values of SNpc were significantly increased in the early-stage PD patients compared with the controls. In late-stage PD patients, QSM values were higher than those of the controls and early-stage PD patients (Guan et al., 2017). Magnetic resonance spectroscopy, relying on the resonance frequencies of protons, could be used to measure the number of biochemical molecules in the brain. The pre-supplementary motor area NAA/Cr was lower in PD patients and correlated negatively with age; however, it did not correlate with the UPDRS score, disease duration, or dopamine equivalents (Camicioli et al., 2007).

Diffusion tensor imaging, an *in vivo* tractography technique, allowed researchers to determine an indirect estimation of brain microstructural integrity in PD patients by analyzing water molecules (Saeed et al., 2017). Sampedro et al. (2019) investigated longitudinal changes in intracortical mean diffusivity (MD) in recently diagnosed and drug-naïve PD patients using a public database (the Parkinson’s Progression Markers Initiative). They found that *de novo* PD patients showed a higher MD value in the frontal and occipital cortices after one year when compared with that of the control subjects, and these changes were correlated with changes in cognitive measures (Sampedro et al., 2019). Reduced fractional anisotropy (FA) of the SN was observed in PD patients when compared with that of control subjects, and the reduced FA of PD patients was greater in the caudal region compared with the rostral region of interest. The sensitivity and specificity of 100% for the diagnosis of PD were obtained when using caudal SN (Vaillancourt et al., 2009).

A functional MRI captures the patterns of coherent spontaneous fluctuations of blood oxygen levels, allowing investigation of functional connectivities and activates (Li et al., 2018). Zhang et al. (2019) observed an increased dynamic amplitude of low-frequency fluctuations in the left precuneus in PD patients, which was positively correlated with disease duration. Machine learning using these features indicated that 80.36% of the patients were correctly diagnosed (Zhang et al., 2019). Another study showed no difference between PD and control participants regarding dynamic functional connectivity of the default mode network or the frontoparietal network with the rest of the brain (Engels et al., 2018). In the late-stage of the disease, PD patients may suffer from the freezing of gait (FOG). The PD patients with FOG exhibited decreased voxel-mirrored homotopic connectivity in the inferior parietal lobe when compared with both PD patients without FOG and healthy controls, so these features could distinguish FOG^ + ^ patients from FOG^−^ patients or healthy controls (Li et al., 2018).

### PET

PET is an *in vivo* functional neuroimaging technique (Matthews et al., 2018). In fluorodeoxyglucose (FDG) PET meta-analyses, glucose hypometabolism was found in the bilateral inferior parietal cortex and left caudate nucleus (Albrecht et al., 2019). FDG-PET could also discriminate PD patients from control subjects and can predict the Hoehn and Yahr stage and UPDRS score, which indicates FDG-PET is an objective, sensitive biomarker of disease stage (Matthews et al., 2018). Another study found that PD patients with rapid eye movement sleep behavior disorder showed widespread reduced binding of ^11^C-MeNER, which was correlated with electroencephalogram slowing, cognitive performance, and orthostatic hypotension (Sommerauer et al., 2018). The tau tangle ligand, ^18^F-AV-1451, binds to neuromelanin in the midbrain. Hansen et al. investigated the value of ^18^F-AV-1451 PET in measuring the concentration of nigral neuromelanin in PD patients and observed a decrease in the ^18^F-AV-1451 signal in the midbrain, which indicated ^18^F-AV-1451 might be the first radiotracer to reflect the loss of pigmented neurons in the SN (Hansen et al., 2016). ^18^F-FP-CIT, a PET radiotracer, was used to reflect dopamine transporters. The dopaminergic depletion in all striatal subregions was negatively correlated with the UPDRS-III score (Park et al., 2019).

### TCS

TCS, a noninvasive and widely available neuroimaging technique, uses ultrasound to estimate the echogenicity of brain tissues *via* the intact cranium. Increased echogenicity of the SN observed by TCS is considered a biomarker of idiopathic PD (Camicioli et al., 2007). Gaenslen et al. (2008) performed a prospective blinded study and found that the sensitivity of TCS at baseline was 90.7% and the specificity was 82.4%, according to endpoint diagnosis, and the positive predictive value and classification accuracy of TCS for idiopathic PD was 92.9% and 88.3%, respectively (Gaenslen et al., 2008). PD patients with an area of the SN echogenicity above a specific threshold value showed an early onset age, and the area of the SN echogenicity with a larger contralateral to the side was related to more severe symptoms (Berg et al., 2001).

## Physiological Biomarkers

Deep brain stimulation (DBS) surgery provides an excellent opportunity to record local field potentials (LFPs) in PD patients, which has provided valuable insight into disease mechanisms and can be used as biomarkers to reflect the syndrome severity. Here we classified these biomarkers based on frequency bands, which will standardize the methodology and simplify the interpretation of these biomarkers.

### Delta Band

PD is a neural degeneration disease, and the patients also have cognition problems. In PD dementia patients, there is strong delta activity in the nucleus basalis of Meynert (NBM) as measured using depth electrodes (Nazmuddin et al., 2018). The same frequency band activity is also elevated in LBs dementia (Gratwicke et al., 2020), which means that this frequency band is a potential biomarker to predict the cognitive decline in PD patients. This frequency band is especially important for NBM mapping (Nazmuddin et al., 2018). The phenomenon also shows that neurophysiology biomarker detection is related to the recording target. Although different targets can detect the same frequency band, a specific target is easy to detect a specific frequency band.

### Theta and Alpha Bands

Several studies have referred to the theta and alpha bands as low-frequency bands (Wojtecki et al., 2017; Mazzoni et al., 2018), but we will discuss these frequency bands together. These frequency bands are related to cognitive processing and motor feedback. An augmentation of low-frequency activity in the subthalamic nucleus (STN) can be observed in conflicting tasks, including economic decision making and moral sentence evaluations (Fumagalli et al., 2011), which indicate that low frequency might be involved in conflict problem judgment. The low-frequency band activity is elevated in the STN after dopamine therapy, which sometimes is accompanied by impulse control disorder (Priori et al., 2004). This phenomenon indicates that a potential dopamine-dependent low-frequency band in the STN may be involved in conflictual and economic decision making. This frequency band is also related to motor feedback. After levodopa intake or DBS, the low-frequency activity is elevated in the STN and GPi, which often correlates with the improvement of some syndromes (Silberstein et al., 2003; Giannicola et al., 2013). Dyskinesia is a side effect of DBS or levodopa, and studies have found that the low-frequency band is elevated in this situation (Alonso-Frech et al., 2006). When combining this information, the low-frequency band is a good biomarker for a hyperkinetic state. A previous study indicated that the LF/beta power ratio was a good neural feedback biomarker to trigger stimulation in adaptive DBS (Giannicola et al., 2012). The low-frequency band is an important biomarker in supplementing the beta band because the beta band is spatially specific. Another important observation is that the alpha band activity is associated with gait. A previous study found the power of alpha oscillation was correlated with gait speed (Thevathasan et al., 2012). One study found that the alpha band power decrease was associated with the probability of freezing (Androulidakis et al., 2008), so this frequency band can be potentially used as a biomarker to regulate gait.

### Beta Band

The beta band is the most popular frequency band used in PD studies. The beta power is elevated in PD patients and decreased after DBS and levodopa intake (Brown et al., 2001). This frequency band is closely related to movement, which decreases with movement initiation and rebounding after movement termination (Canessa et al., 2016). The beta band is a good biomarker to trigger stimulation in adaptive DBS because beta power is positively correlated with rigidity and bradykinesia in PD patients (Brittain and Brown, 2014). The beta power suppression is positively related to the improvement of motor impairment (Kühn et al., 2009). One previous study used the beta power as a biomarker in adaptive DBS, showing that the clinical effect was better than with conventional DBS (Beudel et al., 2018). The constancy of beta power has also contributed to adaptive DBS, and a study found that the long-term beta power could be recorded safely using Medtronic PC + S devices (Neumann et al., 2017).

The beta band is further divided into high beta and low beta bands. The low beta band is closely related to clinical syndromes like bradykinesia and rigidity (Tsiokos et al., 2017). Compared with the low beta band, the high beta band is more related to long-distance (interregional) coupling. The high-frequency oscillation of the cortical and high beta of the STN phase-amplitude coupling is prokinetic (Ozturk et al., 2020). This biomarker might be closely related to the hyper-direct pathway, because of shorter time delays in the high beta band and cortical signal-coupling delays, when compared with the low beta band (Alexander and Crutcher, 1990). This biomarker is more likely physiological rather than pathological during PD because it is related to force generation (Florin et al., 2013a).

As an important biomarker, the beta band has many other applications. The power of the low beta band increases when FOG appears (Chen et al., 2019). Notably, the increased 18 Hz activity is specifically observed in the STN during freezing (Storzer et al., 2017). This biomarker also can be used to evaluate the bilateral hemisphere “crosstalk” network in the FOG syndrome; the low beta band coherence between two hemispheres is related to the possibility of FOG (Anidi et al., 2018). The low beta band can also be used to classify the PD phenotypes because this frequency band is increased in a kinetic rigid group rather than in tremor dominant patients (Trager et al., 2016). This biomarker is suitable for optimizing lead implantation and postoperative programming. Good beta-band activity and strong STN-cortex phase-amplitude coupling were found to be located in the dorsolateral STN. This feature can be used in intraoperative guidance (Zaidel et al., 2010). The beta band can also serve as a feedback biomarker to automatically screen possible optimal contacts. A previous study reported that the contact pair with maximal beta power in the STN was very likely to be the optimal contact, which will have a wide therapeutic window and which will lead to the best symptom control (Tinkhauser et al., 2018).

### Gamma Band

The gamma-band is a prokinetic biomarker, which is elevated at the onset of movement (Florin et al., 2013b), and is completely different from the beta band. Another interesting phenomenon is that gamma activity shows an augmentation after administration of levodopa (Elben et al., 2018). One previous study combining the magnetoencephalogram and LFPs also supported the prokinetic features of gamma oscillation, which revealed a strong coherence in the gamma band between the STN and cortical areas during voluntary movement initiation (Litvak et al., 2012). The above features make the gamma band a suitable biomarker to add for adaptive DBS. During imagery gripping without actual action, we can still observe the enhancement of gamma oscillation (Fischer et al., 2017), which indicates that gamma power is suitable for the decoding movement force in PD patients.

The above frequency bands can be used as biomarkers to reflect the severity of the motor syndrome or the cognitive decline in PD patients. These biomarkers have close relationships with clinical parameters. In-depth analyses of these biomarkers will also aid in the development of future adaptive DBS for PD.

## Discussion

The mechanism of PD pathogenesis remains elusive, and conventional drug or surgery therapies have been unsatisfactory. Early diagnosis of PD is particularly important, but the classical clinical symptoms of PD occur in the middle or advanced stages of the disease and have many common clinical features with some other neurodegenerative diseases. Novel, reliable, sensitive, and early measured biomarkers are essential for early and accurate diagnosis, to predict and monitor disease occurrence and progression. The development of neuroimaging techniques can be used to detect early changes in PD patients and assess the progression of the disease, which should make it a reliable biomarker for PD, by evaluating the potential local structure, cell ultrastructural, perfusion pattern changes, and molecular alterations in PD patients. The emerging neurophysiological biomarkers sensitively reflect the specific syndromes in PD patients, which is well suited in developing the adaptive DBS and predicting the prognosis. Genetics has been associated with susceptibility to PD. To date, at least 20 risk genes have been confirmed to be involved in familial PD, so they may be born with or persist in patients for decades before the onset of clinical symptoms. Consequently, the positive genes or encoding related proteins in peripheral blood also can become potential biomarkers for the diagnosis of PD. Neuroinflammation is a feature of PD pathology and has received increasing attention. Microglial activation, and cytokines, complement or ROS in plasma, serum, or CSF also can provide diagnoses and reflect disease progression, and have the potential to be early diagnostic markers for PD. However, because PD is a complex and progressive neurodegenerative disease, the roles of various biomarkers are distinct at different stages of the disease process. Also, the profiles of biomarkers vary from person to person. A single biomarker may not be sufficient for early diagnostics and prediction of disease progression with adequate stability, sensitivity, or specificity, so a comprehensive biomarker dataset including clinical symptoms, neuroimaging, neurophysiology, and biochemical and genetic information should be used for early diagnostics to predict disease progression.

Also, α-Syn is the key protein associated with PD and is central to the pathogenesis of this disease. The α-Syn is both a promising candidate for a prognostic marker of PD and may act as a trigger during PD development. However, misfolded α-Syn forms oligomers and aggregates that are believed to be toxic for neurons, and α-Syn itself is the primary trigger of the immune response in PD, with its aggregates being sufficient to induce the inflammation. Neuroinflammation is a feature of PD pathology, which might also play a pivotal role in disease pathogenesis, due to chronic microglial activation, the release of cytokines and chemokines, component activation, and ROS. Other PD risk genes have also been implicated in α-Syn aggregation to varying degrees, such as *GBA*, *LRRK2*, *Parkin*, and *PINK1*. Misfolded and aggregated α-Syn is indeed a pathogenic event in PD. However, the actual factors that cause abnormal misfolding and aggregation of α-Syn leading to PD are not straightforward. Moreover, determining how α-Syn is released and taken up by neurons, and how it grows new aggregates in a healthy cell will facilitate a better understanding of the role of α-Syn in PD, and even its potential to act as a biomarker in support of improved clinical diagnoses. The development of biomarkers to help with early differential diagnosis and to predict disease progression from its earliest stage is of major importance, both for research and therapeutic development. Further understanding of the role of α-Syn in PD pathogenesis will provide novel insights for disease diagnosis and therapy, especially at the early stages of PD. The development of novel therapies against neurodegenerative disorders requires the ability to detect their early presymptomatic manifestations to enable treatment before irreversible cellular damage occurs.

## Author Contributions

TD and JZ conceived the manuscript. TD, LW, WL, GZ, and YC wrote the manuscript. All authors contributed to the article and approved the submitted version.

## Conflict of Interest

The authors declare that the research was conducted in the absence of any commercial or financial relationships that could be construed as a potential conflict of interest.
